# Regional Differences in Medical Costs of Chronic Kidney Disease in the South Korean Population: Marginalized Two-Part Model

**DOI:** 10.2196/39904

**Published:** 2023-03-30

**Authors:** Minah Park, Choa Yun, Jae Hong Joo, Soo Hyun Kang, Sung Hoon Jeong, Chung-Mo Nam, Eun-Cheol Park, Yoondae Han, Sung In Jang

**Affiliations:** 1 Institute of Health Services Research Yonsei University Seoul Republic of Korea; 2 Department of Biostatistics and Computing College of Medicine Yonsei University Seoul Republic of Korea; 3 Department of Health Informatics and Biostatistics Graduate School of Public Health Yonsei University Seoul Republic of Korea; 4 Department of Preventive Medicine College of Medicine Gachon University Incheon Republic of Korea; 5 Department of Rehabilitation Medicine Seoul National University Hospital Seoul Republic of Korea; 6 Department of Preventive Medicine College of Medicine Yonsei University Seoul Republic of Korea; 7 Division of Colorectal Surgery, Department of Surgery Severance Hospital Yonsei University College of Medicine Seoul Republic of Korea

**Keywords:** chronic kidney disease, cost analysis, medical expenses, medically vulnerable regions, kidney, public health, cost, economic, chronic disease, insurance, regional, longitudinal model

## Abstract

**Background:**

There are regional gaps in the access to medical services for patients with chronic kidney disease (CKD), and it is necessary to reduce those gaps, including the gaps involving medical costs.

**Objective:**

This study aimed to analyze regional differences in the medical costs associated with CKD in the South Korean population.

**Methods:**

This longitudinal cohort study included participants randomly sampled from the National Health Insurance Service-National Sample Cohort of South Korea. To select those who were newly diagnosed with CKD, we excluded those who were diagnosed in 2002-2003 and 2018-2019. A total of 5903 patients with CKD were finally included. We used a marginalized two-part longitudinal model to assess total medical costs.

**Results:**

Our cohort included 4775 (59.9%) men and 3191 (40.1%) women. Of these, 971 (12.2%) and 6995 (87.8%) lived in medically vulnerable and nonvulnerable regions, respectively. The postdiagnosis costs showed a significant difference between the regions (estimate: –0.0152, 95% confidence limit: –0.0171 to –0.0133). The difference in medical expenses between the vulnerable and nonvulnerable regions showed an increase each year after the diagnosis.

**Conclusions:**

Patients with CKD living in medically vulnerable regions are likely to have higher postdiagnostic medical expenses compared to those living in regions that are not medically vulnerable. Efforts to improve early diagnosis of CKD are needed. Relevant policies should be drafted to decrease the medical costs of patients with CKD disease living in medically deprived areas.

## Introduction

Chronic kidney disease (CKD) has been known as a causative factor for early death and is being recognized as a global health problem [1]. In the United States, 1 in 7 adults are known to have CKD, and recent data show that the number of deaths due to CKD in the past 2 decades has increased. With the increased incidence of CKD, the cost of health care has increased substantially [2]. The most severe phase of CKD, which is end-stage renal disease (ESRD), has cost Medicare US $32.9 billion [2], with earlier stages costing Medicare approximately US $48 billion in 2010 [3]. This phenomenon is also evident in South Korea. In South Korea, the number of patients with CKD has increased by 8.7% annually over the last 5 years (from 2013 to 2017), and CKD was estimated to affect approximately 4.6 million patients as of 2017. The annual medical fee per patient with CKD is 8,361,000 won (US $6367.04), and the annual total medical cost of these patients exceeds 1.7 trillion won (1.2 billion dollars), which makes the per-patient treatment cost for CKD the highest compared with that for other diseases, such as dementia or cancer [4].

In the case of chronic diseases, the effective implementation of medical care, management, and prevention of these diseases are important for social integration [5]. However, in South Korea, despite the need for more medical help in provincial areas, resources and medical expenditure are concentrated in big cities. As of 2009, Seoul was home to 27.6% of the country’s medical specialists and 52.4% of its physicians and dentists. In addition, medical expenditure is concentrated in the capital region. As of 2008, Seoul accounted for 26.9% of all insured medical bills, while regions outside Seoul and the capital region accounted for 36.2% and 14.5% of insured medical bills, respectively [6]. Patients with CKD living in rural areas have a higher risk of morbidity, hospitalization, and mortality compared to those living in urban areas [7]. In addition, rural residents are likely to have low income levels and need to travel farther to seek medical care, which can result in further disparities in CKD care [8]. As South Korea becomes an aging society, rural areas are observed to be aging more rapidly than urban areas [9]. Considering that age is an important factor for CKD [10], its treatment is even more challenging in rural areas with high aging rates. Since there are clear economic and regional gaps in access to services in the health system, it is necessary to make efforts to improve access to essential medical services for CKD.

Kim et al [11] studied the cost-utility data among patients with ESRD, although their analysis was limited due to the data being collected from a specific hospital. Another study [12] on the economic burden of CKD in South Korea used a national sample cohort; however, they found no regional differences. Therefore, our study aimed to estimate the regional differences using the position value for relative composite index of the medical costs of CKD in the South Korean population. We used the data available from the Korean Health Insurance Service.

## Methods

### Data and Study Population

The data for this study were obtained from the National Health Insurance Service- National Sample Cohort (NHIS-NSC). The NHIS-NSC data were collected by random sampling of medical claims, covering 2% of the South Korean population. The period during 2002 to 2003 was designated as a washout period to account for the effects of other existing diseases that might influence the results associated with the hypothesized relationship. To select those who were newly diagnosed with CKD, we excluded those who were diagnosed with CKD in 2002-2003 and 2018-2019. From these data, we extracted 10,019 cases diagnosed with CKD (International Classification of Diseases, 10th revision; code: N18). After excluding patients who were diagnosed with CKD during the excluded period, a total of 7966 individuals were included in the final study (vulnerable regions: n=971, 12.2%; nonvulnerable regions: n=6995, 87.8%).

### Ethical Consideration

This study was reviewed and approved by the International Review Board of Yonsei University’s Health System (Y-2020-0031) and adheres to the tenets of the Declaration of Helsinki. The NHIS-NSC data do not contain any identifying information; thus additional approval was not required.

### Variables

The variable of interest in this study was the region. Position value for relative composite (PARC) indicators were used to categorize the health care level by region in South Korea; the analysis methods have been explained in detail in previous studies [13-18]. PARC is an objective indicator that identifies the relative health care level of a location compared with other regions. The PARC value ranges from −1 to 1, with a value of 1 as the best, 0 as the average, and −1 as the worst value, compared with the mean value of the entire region; the closer the value is to −1, the lower the health care level in the region compared with the average; on the other hand, the closer the value is to 1, the higher the level is compared with the average. In this study, when the PARC value was less than −0.33, the region was classified as a medically vulnerable region.

The dependent variable of this study was the total medical cost. Based on the time of diagnosis, the medical expenses were calculated monthly for 24 months before diagnosis and 24 months after diagnosis. Medical expenses before death were also included.

In addition, analyses that included sex, age, income, and social security type as independent variables were performed. Social security was categorized into health insurance (eg, corporate or regional) and medical aid. One of the social security systems in South Korea, National Health Insurance Service (NHIS), covers the entire population except for medical aid beneficiaries. Since the South Korean government provides insurance for the poor, it offers a medical aid program to people unable to pay for their own health care coverage. The medical aid program beneficiaries are financed by both the local and central government and are a part of the South Korean public assistance system [19].

### Statistical Analyses

#### Marginalized Two-Part Longitudinal Model

To overcome the limitations posed by a conventional two-part model, we used a marginalized two-part (MTP) longitudinal model that directly parameterizes the marginal mean of *Y_ij_* with covariates [20]. The MTP model has the same two-part structure as the conventional model, but instead of parametrizing the model with a conditionally positive-valued log-scale location parameter *μ_ij_*, it models the marginal mean *ν_ij_* that is conditional expectation value of *Y_ij_* on *b_i_* for medical users and nonusers combined. Thus, we take the following equation:



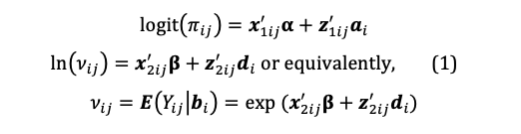



We redefined *b_i_* to now have *b'_i_* vector that includes a*'_i_* and *d'_i_*, the random effects are assumed to jointly follow a multivariate normal distribution; this allows cross-part correlation among random effects. Under the MTP parameterization, *β_k_* is the incremental effect in the log of the overall mean, *E*(*Y_ij_* |*b_i_*), that comprises the entire population, including both medical users and nonusers, *E*(*Y_ij_* |*b_i_*), and corresponds to a unit increase in the *k*th covariate, *x_2kij_*.

#### Model Details

The percentage of the study population with 0 expenditure over the study period was 29%, creating the need for a two-part model. To assess the effect of the regional difference on medical costs, we fit the MTP model with explanatory variables in the binary and overall mean components. In addition, we used segmented regression analysis for powerful estimation of intervention effects in an interrupted time series. The full model was constructed as follows:



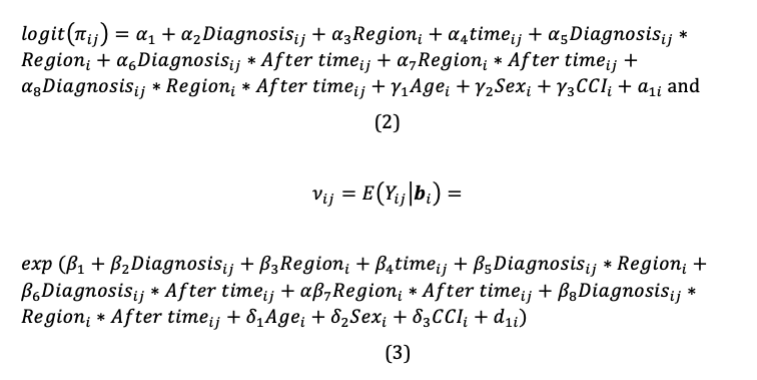



In the above, 0 ≤ *time_ij_* ≤ 48 and 0 ≤ *After time_ij_* ≤ 24.

We fit the MTP model using the %MTPmle SAS macro program that correctly estimates model parameters through the likelihood function and can be maximized using SAS PROC NLMIXED. In addition, we chose a model type that used a continuous distribution in the second part of the MTP model: the gamma distribution.

## Results

We analyzed the descriptive statistics of the medical costs of CKD by region (Table 1). The people living in the vulnerable region comprised 12.2% (971/7966) of participants, and those in the nonvulnerable region comprised 87.8% (6995/7966).

We analyzed the summary statistics for the semicontinuous outcome at each time point (Table 2). After the diagnosis of CKD, significant differences were found in region, sex, income, and Charlson comorbidity index between the groups.

We calculated the overall means and 95% confidence limits of the MTP model parameters (Table 3). There was a significant difference in difference in pre- and postdiagnosis medical costs between the regions (estimate: –0.0152, 95% CI 0.0171 to –0.0133).

We mathematically expressed the MTP model–estimated slope effects of differences in diagnosis and region (Table 4).

We used the MTP model to estimate the effect of region on medical expenses over time (Table 5). The first 2 years are the estimates, and the following years are predictions. The difference in medical expenses between vulnerable and nonvulnerable regions increased every year.

**Figure 1:**
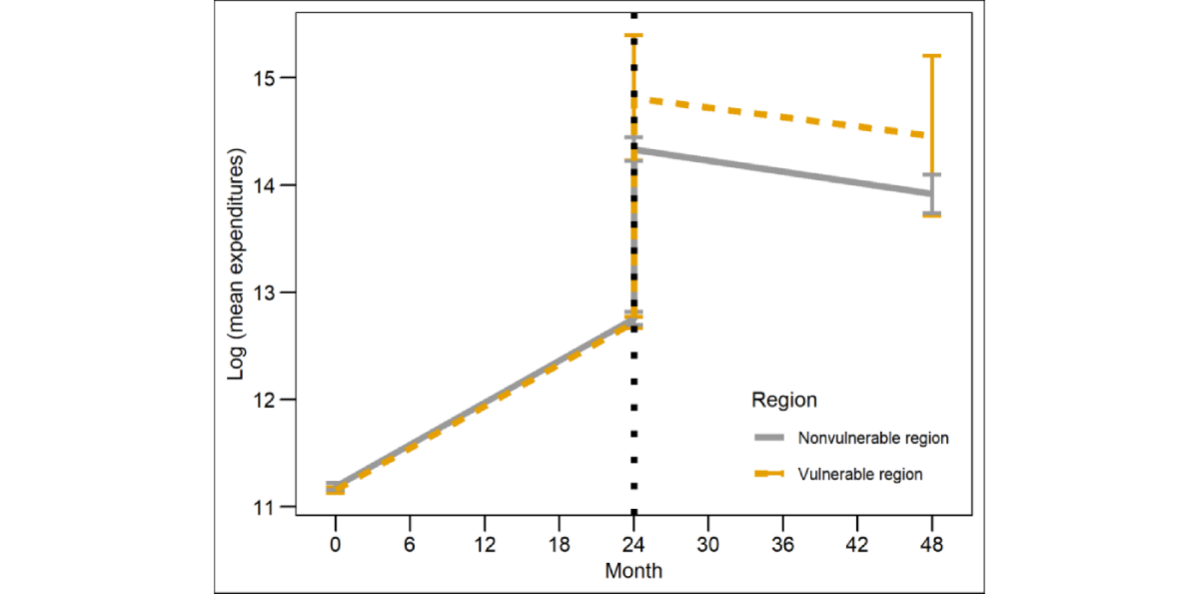
Model of the estimated log (mean expenditures) and 95% CIs for the chronic kidney disease costs.

The overall mean of the values with log scale in Table 2 was calculated (Figure 1). Before the CKD diagnoses, there was a significant difference in the slope or intercept. There was also a clear difference in both the slope and intercept after the diagnosis.

**Table 1:** Descriptive statistics of the medical costs of chronic kidney disease analyzed by region.

Characteristics			Region							*P* value
			Total (N=7966, 100), n (%)		Vulnerable regions (n=971, 12.2), n (%)		Nonvulnerable regions (n=6995, 87.8), n (%)			
**Sex**										.40
	Male	4775 (59.9)		570 (58.7)		4250 (60.1)				
	Female	2790 (40.1)		401 (41.3)		2790 (39.9)				
**Age (years)**										<.001
	<30	559 (7.0)		56 (5.8)		503 (7.2)				
	30-39	742 (9.3)		79 (7.5)		669 (9.3)				
	40-49	1303 (16.4)		124 (9.5)		1179 (16.9)				
	50-59	1769 (22.2)		215 (22.1)		1554 (22.2)				
	60-69	2451 (30.8)		343 (35.3)		2108 (30.1)				
	70-79	1106 (13.9)		154 (15.9)		952 (13.6)				
	>80	36 (0.5)		6 (0.6)		30 (0.4)				
**Coverage type**										.15
	NHI^a^, employed, or self-employed	3799 (47.7)		442 (45.5)		3357 (48)				
	Medical aid	4167 (52.3)		529 (54.5)		3638 (52)				
**Income**										<.001
	Low	1094 (13.7)		132 (13.6)		962 (13.8)				
	Middle	3534 (44.4)		488 (50.3)		3046 (43.6)				
	High	3338 (41.9)		351 (36.2)		2987 (42.7)				
**CCI^b^**										<.001
	0	1645 (20.7)		163 (16.8)		1482 (21.2)				
	1	2300 (28.9)		266 (27.4)		2034 (29.1)				
	>2	4021 (50.5)		542 (55.8)		3479 (50)				

^a^NHI: National Health Insurance.

^b^CCI: Charlson comorbidity index.

**Table 2:** Summary statistics for the semicontinuous outcome (expenditures) at each time point. Monthly expenditures were calculated by converting South Korean won to US dollars.

Characteristics		Overall expenditures			Expenditures before diagnosis		Expenditures after diagnosis	
		Mean (SD)		*P* value	Mean (SD)	*P* value	Mean (SD)	*P* value
**Region**				.12		.67		.02
	Vulnerable region	313 (411)			209 (303)		416 (682)	
	Nonvulnerable region	291 (390)			214 (379)		368 (586)	
**Sex**				.10		.76		.05
	Male	288 (382)			212 (377)		363 (570)	
	Female	303 (408)			215 (361)		390 (639)	
**Age (years)**				.01		<.001		.13
	<30	266 (414)			170 (426)		362 (614)	
	30-39	266 (420)			162 (452)		370 (637)	
	40-49	294 (441)			195 (427)		393 (666)	
	50-59	311 (411)			230 (353)		392 (617)	
	60-69	306 (369)			236 (339)		375 (573)	
	70-79	274 (320)			216 (294)		332 (502)	
	>80	250 (254)			237 (296)		262 (361)	
**Coverage type**				.11		.35		.13
	NHI^a^, employed, or self-employed	286 (392)			209 (365)		363 (597)	
	Medical aid	300 (394)			217 (376)		384 (599)	
**Income**				.02		.52		.01
	Low	318 (384)			225 (372)		411 (598)	
	Middle	298 (396)			213 (356)		382 (609)	
	High	282 (392)			210 (385)		353 (586)	
**CCI^b^**				<.001		<.001		<.001
	0	173 (311)			108 (278)		237 (472)	
	1	265 (385)			177 (316)		354 (622)	
	>2	359 (413)			278 (418)		441 (621)	

^a^NHI: National Health Insurance.

^b^CCI: Charlson comorbidity index.

**Table 3:** Overall means and 95% CIs of the marginalized two-part model parameters.

Characteristics		Parameter	Estimate	95% confidence limit
**Binary component**				
	Intercept	*α*_1_	–0.4169	(–0.4501, –0.3835)
	Diagnosis	*α*_2_	–0.03515	(–0.0668, –0.0035)
	Region	*α*_3_	0.1875	(0.1533, 0.2218)
	Time	*α*_4_	0.0260	(0.0244, 0.0275)
	Diagnosis×region	*α*_5_	–0.1124	(–0.1839, –0.0408)
	Diagnosis×after time	*α*_6_	–0.0888	(–0.0910, –0.0867)
	Region×after time	*α*_7_	–0.0151	(–0.0172, –0.0130)
	Diagnosis×region×after time	*α*_8_	0.0112	(0.0091, 0.0133)
	Age	*γ*_1_	0.0116	(0.0161, 0.0171)
	Sex	*γ*_2_	–0.0816	(–0.0962, –0.0671)
	CCI^a^	*γ*_3_	0.3348	(0.3257, 0.3440)
**Overall mean component**				
	Intercept	*β*_1_	11.1894	(11.1561, 11.2226)
	Diagnosis	*β*_2_	0.0123	(–0.0119, 0.0366)
	Region	*β*_3_	–0.0356	(–0.0611, –0.0101)
	Time	*β*_4_	0.0652	(0.0641, 0.0664)
	Diagnosis×region	*β*_5_	0.0884	(0.0332, 0.1435)
	Diagnosis×after time	*β*_6_	–0.0825	(–0.08430, –0.0807)
	Region×after time	*β*_7_	0.0178	(0.0159, 0.0197)
	Diagnosis×region×after time	*β*_8_	–0.0152	(–0.0171, –0.0133)
	Age	*δ*_1_	–0.0002	(–0.0006, 0.0003)
	Sex	*δ*_2_	–0.0635	(–0.0762, –0.0507)
	CCI	*δ*_3_	0.3734	(0.3650, 0.3818)

^a^CCI: Charlson comorbidity index.

**Table 4:** Marginalized two-part model–estimated slope effects of differences in diagnosis and region (mathematical expression).

Characteristics	Before	After	After-before
Vulnerable region	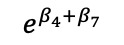	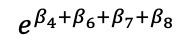	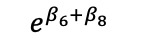
Nonvulnerable region	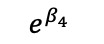	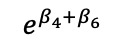	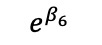
Difference of regions	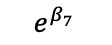	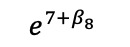	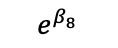

**Table 5:** Marginalized two-part model–estimated effects of region over time.

	1 year	2 years	3 years^a^	4 years^a^	5 years^a^
Difference (%)	0.26	0.52	0.78	1.05	1.31

^a^The data used in the marginalized two-part model analysis are for 2 years before and 2 years after diagnosis. The estimated effects at 3, 4, and 5 years are predicted values.

## Discussion

In this study, we examined the differences in medical costs of CKD among the regions of South Korea. The study results demonstrated that there were differences in postdiagnosis medical costs for patients with CKD depending on the region. Patients living in medically vulnerable areas had higher medical costs than patients living in nonvulnerable areas.

These findings are consistent with those of previous studies on chronic obstructive pulmonary disease, as our analysis on this disease (Multimedia Appendix 1), which found that deprivation of health care resulted in an increase in medical costs, emergency health care use, and mortality [21]. As shown in our results, people in medically vulnerable areas were likely to spend more in terms of medical costs. This could be because people living in vulnerable areas discover their symptoms only when conditions have become severe, which results in higher medical costs. Since social deprivation has been found to affect the development of a kidney condition, it is likely that the stage of CKD may worsen [22]. Diagnosis of CKD tends to happen at later stages, resulting in treatment delay and poorer outcomes [23]. Many studies, including those conducted in Canada [24], have found that rural residents with CKD are likely to have less access to specialty care and are provided with low-quality care, which results in a higher prevalence of severe stages of CKD in these areas. As previous studies have shown [3,25], more severe symptoms cause a rapid increase in medical expenses.

Geographical location plays an important role in the treatment of CKD. Some treatments, such as hospital-based hemodialysis, may not be feasible in rural areas. For patients who are dependent on ambulance service, transportation also becomes an important issue [26]. There are many indicators, such as Index Multiple Deprivation, that are used in the United Kingdom to evaluate deprivation within a geographic area [27]. The PARC indicators measure many aspects of the health care level by region in South Korea, rather than simply classifying regions as rural or urban; therefore, they might provide more accurate indicators for evaluation of the actual deprivation of health care regarding the medical cost of CKD.

Since there is an increase in postdiagnosis medical expenses for CKD in vulnerable areas, necessary policies should be implemented to lower the burden of this condition. In addition, efforts to increase early diagnosis of CKD are needed. Currently, most cases of CKD are detected during the course of treating other health problems rather than because of any CKD symptoms. Often, the early stages of CKD show no symptoms, and discovery is made only when conditions become severe. As shown previously [3], early detection of CKD is needed to lower the medical expenses of the patient.

Our study has some important limitations. First, we were not able to perform a random slope analysis in the study because SAS software does not currently provide the necessary program. In future studies, using another statistical program would avoid this issue. Second, since the data set is a collection of medical-claim bills, it is highly likely that the actual number of patients with CKD and their actual burdens are higher than the reported numbers. In general, the number of patients with CKD can be said to represent a pyramid with ESRD at its peak, although the number of patients with CKD based on their treatment performance with health insurance and medical benefits shows the opposite picture [28]. In addition, data on socioeconomic costs that could affect medical expenses, such as transportation, privately hired caregivers, private health insurance, and health supplements, were not available for this study.

However, there are some major strengths to our study. To the best of our knowledge, this study is the first to investigate the regional differences in the medical costs of CKD in the South Korean population using a TPM model. In addition, since all South Korean citizens are obligated to enroll in the NHIS, the NHIS data sets provide nationally representative data.

Our findings suggest that patients with CKD living in medically vulnerable regions are more likely to have increased postdiagnosis medical expenses compared with those living in medically nonvulnerable regions. Over time, the differences in medical expenses are likely to increase substantially. Policies are needed to decrease the medical bills of patients with CKD living in medically deprived areas.

## Appendix

Multimedia Appendix 1 Overall means and 95% CIs of the marginalized two-part model parameters for chronic obstructive pulmonary disease.

## Abbreviations

CKD: chronic kidney disease
ESRD: end-stage renal disease
MTP: marginalized two-part
NHIS-NSC: National Health Insurance Service-National Sample Cohort
NHIS: National Health Insurance Service
PARC: position value for relative composite

## Data Availability

All data are available in the database of the Korean National Health Insurance Sharing Service and can be accessed upon reasonable request.
