# Supplementary material for: Regional Differences in Medical Costs of Chronic Kidney Disease in the South Korean Population: Marginalized Two-Part Model
Source: JMIR Public Health Surveill. 2023 Mar 30;9:e39904. doi: 10.2196/39904 (PMC10131872; doi:10.2196/39904)
Supplement: Multimedia Appendix 1 [file publichealth_v9i1e39904_app1.docx]

**Multimedia**  **Appendix 1:**

**Overall means and 95% confidence intervals of the MTP model parameters for chronic obstructive pulmonary disease (COPD)**

|  | **Parameter** | **Estimate** | **95% Confidence Limit** |
| --- | --- | --- | --- |
| **Binary component** |  | | |
| Intercept | $\alpha_{1}$ | -0.8953 | (-0.9092, -0.8814) |
| Diagnosis | $\alpha_{2}$ | 0.2093 | (0.1952, 0.2233) |
| Region | $\alpha_{3}$ | 0.1034 | (0.0914, 0.1153) |
| Time | $\alpha_{4}$ | 0.0257 | (0.0251, 0.0264) |
| Diagnosis$\times$Region | $\alpha_{5}$ | -0.0319 | (-0.0588, -0.0050) |
| Diagnosis$\times$After Time | $\alpha_{6}$ | -0.0002 | (-0.0010, -0.0007) |
| Region$\times$After Time | $\alpha_{7}$ | -0.0639 | (-0.0649, -0.0630) |
| Diagnosis$\times$Region$\times$After Time | $\alpha_{8}$ | 0.0002 | (0.0010, 0.0007) |
| Age | $\gamma_{1}$ | 0.0209 | (0.0207, 0.0211) |
| Sex | $\gamma_{2}$ | -0.3511 | (-0.3575, -0.3446) |
| Charlson comorbidity index | $\gamma_{3}$ | 0.4777 | (0.4730, 0.4824) |
| **Overall mean component** |  | | |
| Intercept | $\beta_{1}$ | 9.7962 | (9.7821, 9.8130 ) |
| Diagnosis | $\beta_{2}$ | 0.2696 | (0.2580, 0.2813) |
| Region | $\beta_{3}$ | -0.0632 | (-0.0736, -0.0529) |
| Time | $\beta_{4}$ | 0.0316 | (0.0310, 0.0322) |
| Diagnosis$\times$Region | $\beta_{5}$ | -0.0644 | (-0.0859, -0.0435) |
| Diagnosis$\times$After Time | $\beta_{6}$ | -0.0571 | (-0.0579, -0.0562) |
| Region$\times$After Time | $\beta_{7}$ | 0.0037 | (0.0031, 0.0044) |
| Diagnosis$\times$Region$\times$After Time | $\beta_{8}$ | 0.0037 | (0.0031, 0.0044) |
| Age | $\delta_{1}$ | 0.0171 | 0.0169, 0.0173) |
| Sex | $\delta_{2}$ | 0.1300 | (0.1242, 0.1358) |
| Charlson comorbidity index | $\delta_{3}$ | 0.5018 | (0.4981, 0.5055) |
